# Effectiveness of Craniectomy Versus Craniotomy in the Management of Acute Subdural Hematoma Patients: A Systematic Review

**DOI:** 10.7759/cureus.75842

**Published:** 2024-12-16

**Authors:** Anna Haymov, Varun Soti

**Affiliations:** 1 Neurosurgery, Lake Erie College of Osteopathic Medicine, Elmira, USA; 2 Pharmacology and Therapeutics, Lake Erie College of Osteopathic Medicine, Elmira, USA

**Keywords:** acute subdural hematoma, craniectomy, craniotomy, mortality, patient outcome surgery, surgical decision-making, traumatic brain injuries

## Abstract

Traumatic brain injuries (TBIs) represent a spectrum of neurological conditions resulting from external forces impacting the head, leading to temporary or permanent impairments in cognitive, emotional, or physical functioning. Acute subdural hematomas (ASDH) are a significant subset of TBIs characterized by the rupture of blood vessels within the subdural space between the brain and the dura mater. Management of ASDH typically involves two primary surgical procedures: craniectomy and craniotomy. This review assessed the efficacy of these surgical approaches in treating patients with ASDH to determine whether one procedure provides superior patient outcomes compared to the other. Furthermore, it aimed to identify factors influencing surgical decisions about the type of procedure. A comprehensive literature search was conducted on ASDH patients undergoing craniotomy and craniectomy, following the Preferred Reporting Items for Systematic Reviews and Meta-Analyses (PRISMA) guidelines. The analysis indicated that craniectomy was associated with higher mortality rates compared to craniotomy. Patients undergoing craniotomy had a statistically heightened likelihood of experiencing residual and rebleeding subdural hematoma. However, the incidence of increased intracranial pressure was significantly more pronounced in craniectomy compared to craniotomy. Follow-up Glasgow Coma Scale (GCS) scores, assessed six months post-surgery, suggested more favorable outcomes for patients who underwent craniotomy, albeit without statistical significance. Furthermore, this systematic review highlighted numerous factors influencing the choice of surgical approach, including the severity of the disease upon admission, patient age, and geographical location. Notably, patients exhibiting a GCS score of less than nine were more likely to be administered craniectomy. Additionally, younger patients, specifically those under 20 years of age with severe injuries, were more frequently subjected to craniectomy. In contrast, neurosurgeons in the United States and several European countries exhibited a preference for craniotomy, whereas craniectomy emerged as the predominant option for ASDH management in the United Kingdom. Ongoing research is essential to ascertain which surgical procedures yield superior patient outcomes within diverse cohorts of ASDH patients. Nonetheless, these findings underscore the critical need for continued investigation to refine surgical strategies and enhance patient outcomes in neurosurgery.

## Introduction and background

Traumatic brain injuries (TBIs) refer to a range of neurological disorders that result from external forces impacting the head, leading to the temporary or permanent impairment of cognitive, emotional, or physical functions [1]. The American Association of Neurological Surgeons (AANS) classifies TBIs based on their severity as mild, moderate, or severe. Mild TBIs, commonly known as concussions, may cause temporary cognitive dysfunction. On the other hand, moderate and severe TBIs may induce profound and long-lasting effects, such as prolonged unconsciousness, memory loss, and personality changes [2].

TBIs represent a pressing global public health issue, with millions of individuals affected annually. In the United States (U.S.), data from the Centers for Disease Control and Prevention reveal that TBIs substantially contribute to emergency department visits, hospitalizations, and long-term disability. According to self-reported data, approximately 15% of high school students in the U.S. experienced one or more sports or recreation-related concussions within the previous 12 months in 2019. In 2020, the number of TBIs-related hospitalizations exceeded 214,000 cases, with approximately 69,350 deaths attributed to TBIs in 2021. It is predicted that TBIs will surpass several diseases as a leading cause of death and disability in the next five years [3].

The mortality rate associated with TBIs varies based on severity. Severe TBIs have a higher likelihood of fatal outcomes. AANS reports that TBIs contribute to a substantial number of deaths annually, making them a significant public health challenge. TBIs can result from various causes, including falls, motor vehicle accidents, sports-related injuries, and assaults [2]. Falls are a leading cause, especially among older adults, while motor vehicle accidents predominantly affect younger populations [4,5]. Understanding diverse etiologies is crucial for developing preventive strategies and enhancing public awareness [6-9].

TBIs manifest in different forms, with primary injuries occurring at the time of impact and secondary injuries developing over time due to cascading processes. Primary injuries may involve contusions, hematomas, and diffuse axonal injuries, while secondary injuries can result from swelling, increased intracranial pressure (ICP), and neurochemical changes [10]. Acute subdural hematoma (ASDH), one of the major types of TBIs, is characterized by the rupture of a blood vessel in the subdural space between the brain and the dura mater. ASDH can cause secondary brain injuries and have a high mortality rate [11,12].

ASDH is known to cause direct damage to the brain tissue at the site of occurrence and lead to the mass effect, resulting in the shifting of the brain due to the accumulation of blood or edema. In approximately 50% of cases, small ASDHs result in the progression of bleeding, leading to elevated ICP. Large ASDHs are typically associated with immediate ICP elevation. The significance of controlling ICP in treating ASDH cannot be overstated. ICP levels are highly correlated with mortality rates, underscoring the importance of pressure management in patient outcomes [13].

To alleviate the mass effect in patients with ASDH, two primary procedures, namely craniectomy and craniotomy, are typically employed. However, it has been observed that even after surgical intervention, more than one-third of patients continue to exhibit elevated ICP values greater than 45 millimeters of mercury (mm Hg). These observations help explain the high mortality rates associated with ASDH and underscore the need for a deeper understanding of treatment methods that can reduce ICP to a normal range of less than 15 mm Hg [14].

Craniectomy and craniotomy are both surgical procedures that involve removing a bone flap from the skull. Craniectomy is typically performed to stabilize ICP, which may require additional surgery to replace the bone flap. This procedure is usually done during hematoma evacuation or to treat cerebral edema and elevated ICP. Occasionally, secondary craniectomies are performed to alleviate pressure following a craniotomy [15].

On the other hand, craniotomy involves the removal and subsequent replacement of a section of the skull to access the brain. While effective, this method may result in an elevated ICP and relies mainly on the mass effect to alleviate the pressure independently. However, alternative craniotomy techniques have emerged that offer the benefit of reducing ICP without requiring a second surgery. These include the hinge craniotomy and the four-quadrant osteoplastic decompressive craniotomy, both of which involve the replacement of the bone flap and allow for outward expansion. These techniques provide a viable solution to reduce ICP and should be considered an alternative to traditional craniectomies [16]. Despite being effective in lowering ICP, craniectomies carry a high risk of postoperative complications, ranging from 10% to 40%. These complications primarily include bone resorption after cranioplasty and infections at the surgical site [17].

Therefore, this review aims to evaluate the efficacy of these two procedures in treating ASDH and ascertain if one procedure yields superior patient outcomes compared to the other. Furthermore, it seeks to identify the factors influencing neurosurgeons’ and healthcare team decisions to prefer one surgical method over the other. Its goal is to provide evidence-based insights, aiding healthcare providers in clinical decision-making and patient-specific treatment strategies. It also seeks to benefit patients and their families by enhancing understanding, empowering them to participate actively in decision-making, and setting realistic expectations. Furthermore, the review guides research efforts by identifying knowledge gaps and stimulating innovation, ultimately contributing to advancements in surgical approaches and patient outcomes in the complex landscape of ASDH management.

## Review

Methods

**Figure 1:**
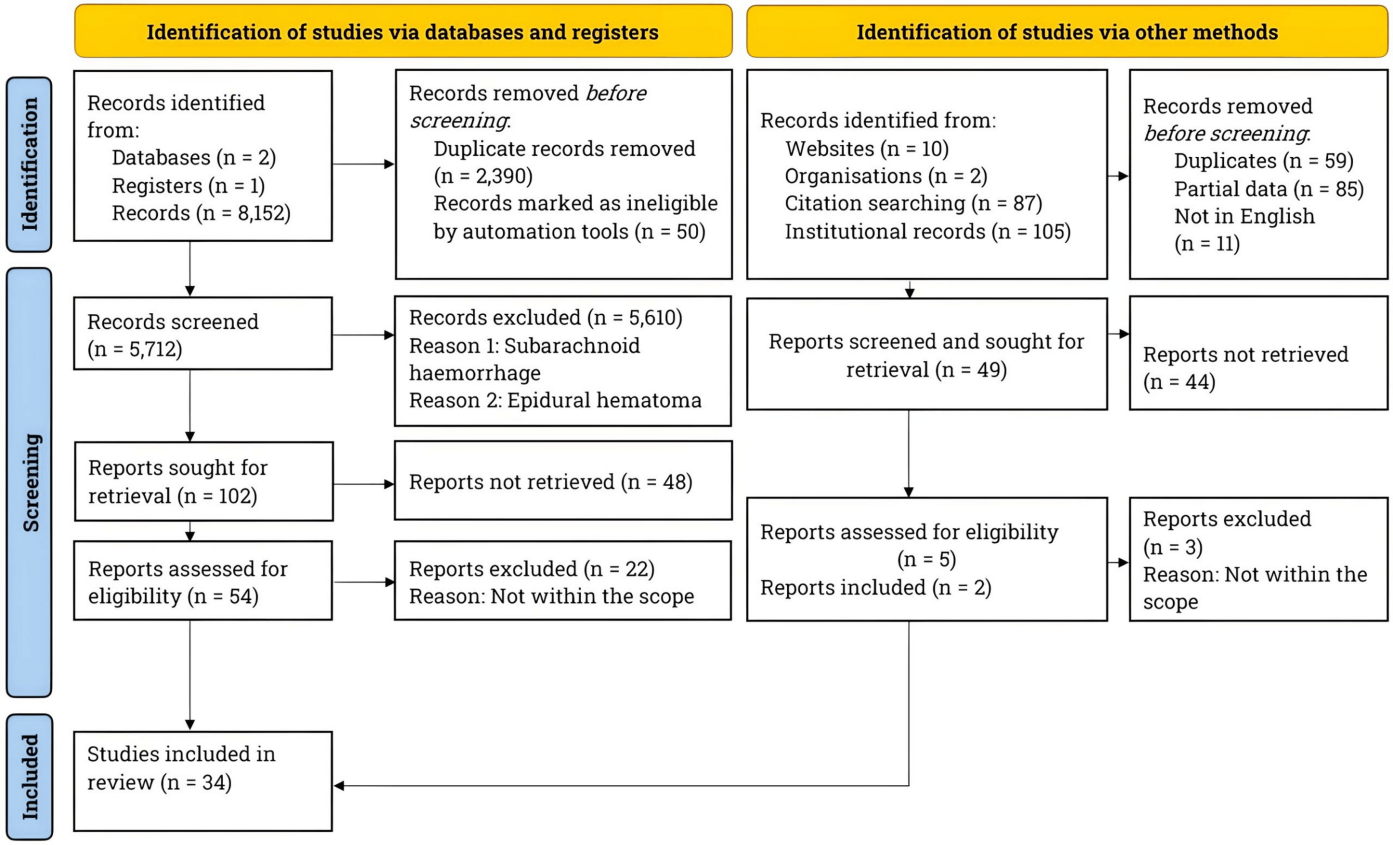
Literature search and study selection process. The flowchart represents the literature search and study selection process. Per PRISMA guidelines, we searched the literature on TBIs and specifically focused on clinical evidence comparing the efficacy of craniectomy and craniotomy in treating patients with ASDH. The search was performed using PubMed, ClinicalTrials.gov, and the Journal of Neurosurgery between January and October 2024. n: Number, ASDH: Acute Subdural Hematoma, PRISMA: Preferred Reporting Items for Systematic Reviews and Meta-Analyses, TBIs: Traumatic Brain Injuries

Per the Preferred Reporting Items for Systematic Reviews and Meta-Analyses (PRISMA) guidelines, we conducted a literature search on PubMed, ClinicalTrials.gov, and the Journal of Neurosurgery between January and October 2024 [18]. Figure 1 depicts the PRISMA flowchart for the literature search and study selection process.

The keywords used in the literature search included “Traumatic Brain Injuries,” “Craniotomy + Subdural Hematoma,” “Craniectomy + Subdural Hematoma,” “Subdural Hematoma + Craniotomy,” “Subdural Hematoma + Craniectomy,” “Craniotomy + Craniectomy + Subdural Hematoma,” and “Craniectomy + Craniotomy + Subdural Hematoma + Clinical Trials + Randomized Controlled Trial.”

The literature search focused on studies involving male and female participants aged 18 years and above with ASDH. All included studies were assigned a level of clinical evidence per previous literature [19]. Table 1 reviews the study inclusion criteria.

**Table 1:** Study selection criteria. This table outlines the criteria for selecting studies in this systematic review. We specifically focused on studies that directly compared craniectomy and craniotomy in the treatment of patients with ASDH. Additionally, our selection encompassed studies that explored various aspects of TBIs, including their epidemiology, etiology, classification, pathophysiology, prognosis, interventions, surgical methods, and public awareness. We prioritized the most relevant and credible studies published in English that met the established selection criteria. ASDH: Acute Subdural Hematoma, BIs: Traumatic Brain Injuries

Inclusion criteria for studies directly comparing the safety and efficacy of craniectomy versus craniotomy in treating ASDH patients	Inclusion criteria for studies focused on TBIs epidemiology, etiology, classification, pathophysiology, prognosis, intervention, other surgical approaches, and public awareness
Randomized controlled trials	Meta-analyses
Non-randomized controlled trials	Systematic reviews
Prospective clinical studies	Narrative reviews
Observational studies	Commentaries
Comparative studies	Opinions
Pilot studies	Pre-clinical studies
Case reports and series	All study types mentioned in left column

This review included 34 studies. Table 2 shows breakdown of the number of studies included in this review, grouped by category.

**Table 2:** Aggregate count of reviewed studies. This table outlines the study distribution in this review, categorized by type. ASDH: Acute Subdural Hematoma, TBIs: Traumatic Brain Injuries

Category	Number of studies
TBIs epidemiology, etiology, and classification	5
TBIs pathophysiology, prognosis, intervention, surgical approaches, and public awareness	12
Method for systematic literature search	2
Clinical evidence of direct comparison of craniectomy versus craniotomy in treating ASDH patients	15
	Total number of studies = 34

Results

Comparison of Mortality Rates Between Craniectomy and Craniotomy

Table 3 presents critical studies included in this review, demonstrating mortality rates between craniectomy and craniotomy in patients with ASDH.

**Table 3:** Critical studies comparing mortality rates between craniectomy and craniotomy in patients with ASDH. This table summarizes the critical studies included in the review, comparing mortality rates between craniectomy and craniotomy in patients with ASDH. The table contains information about sample size, patients’ age, and post-surgical follow-up period. The findings of each study are summarized, highlighting the mortality rates for both procedures. %: Percentage, ASDH: Acute Subdural Hematoma, p: Probability Value

Authors	Level of evidence	Sample size	Patients’ mean/median age	Post-surgical follow-up	Type of surgery	Findings
Ruggeri et al. (2022) [20]	II	94	60 years	12 months	Craniectomy and Craniotomy	The in-hospital mortality rate, 53.2%, remained consistent between craniectomy and craniotomy procedures. However, in patients who survived, craniectomy resulted in better neurological outcomes compared to craniotomy (p = 0.009).
Ahmed et al. (2021) [21]	II	2,370	38 years (craniectomy) and 49 years (craniotomy)	No follow-ups were reported	Craniectomy and Craniotomy	The in-hospital mortality rate after surgery was 37-43%. There was no significant difference between the mortality rates for craniectomy and craniotomy patients (p = 0.10).
Tsermoulas et al. (2016) [22]	III	99	46 years (craniectomy and craniotomy)	6 months	Craniectomy and Craniotomy	Craniotomy resulted in remarkably better patient outcomes than craniectomy (p = 0.037).
Vilcinis et al. (2017) [23]	II	643	54.52 years (craniectomy) and 60.34 years (craniotomy)	Patient follow-ups were not reported	Craniectomy and Craniotomy	The in-hospital mortality rate was significantly higher following craniectomy (54%) compared to craniotomy (20%), p < 0.001.
Woertgen et al. (2006) [24]	II	180	55.2 years (craniectomy and craniotomy)	Between 4 and 132 months	Craniectomy and Craniotomy	The mortality rate was higher in patients who underwent craniectomy (53%) than in those who underwent craniotomy (32.3%). At follow-ups, there was no statistically significant difference in neurological outcomes of patients who underwent the two surgical procedures.
Li et al. (2012) [25]	II	91	45 years (craniectomy) and 59 years (craniotomy)	6 months	Craniectomy and Craniotomy	The mortality rate among patients who underwent craniectomy was slightly higher (38%) than those who had craniotomy (32%), although this difference was not statistically significant (p = 0.65). Similarly, there was no significant difference in the neurological outcomes of patients who had craniectomy and those who had craniotomy (p = 0.83) during follow-up.
Vankipuram et al. (2020) [26]	I	115	38.5 years (craniectomy and craniotomy)	6 months	Craniectomy and Craniotomy	The mortality rate was 37.9% and 45.4% in patients who underwent craniectomy and craniotomy, respectively, with a p-value of 0.6. There was no statistically significant difference in the patients’ favorable neurological outcomes between those who underwent craniectomy and craniotomy at follow-up (p = 0.74).
Rush et al. (2016) [27]	II	1,940	49.5 (Craniectomy) and 68.9 (Craniotomy)	Patient follow-ups were not reported	Craniectomy and Craniotomy	The in-hospital mortality rate was higher in patients who underwent craniectomy (35%) than in those who underwent craniotomy (10.9%), p < 0.0001.
Shibahashi et al. (2020) [28]	II	1,028	63 (Craniectomy) and 64 (Craniotomy)	Patient follow-ups were not reported	Craniectomy and Craniotomy	The in-hospital mortality rates were similar between craniectomy (39.1%) and craniotomy (41.6%) procedures (p > 0.05).
Chen et al. (2011) [29]	II	102	41.2 (Craniectomy) and 47.4 (Craniotomy)	12 months	Craniectomy and Craniotomy	The in-hospital mortality rate was significantly higher after a craniectomy (23.3%) compared to a craniotomy (7.1%), p = 0.04.
Ahmed et al. (2020) [30]	II	1,036	39.6 (Craniectomy) and 38.3 (Craniotomy)	Patient follow-ups were not reported	Craniectomy and Craniotomy	There were no instances of in-hospital mortality.
Ran et al. (2024) [31]	II	138	47.5 (Craniectomy) and 68.9 (Craniotomy)	12 months	Craniectomy and Craniotomy	The in-hospital mortality rate following craniectomy was 48.4%, which was notably higher compared to craniotomy at 26.3% (p = 0.007).
Kwon et al. (2016) [32]	III	46	65.8 (Craniectomy) and 63.4 (Craniotomy)	6 months	Craniectomy and Craniotomy	There were no instances of in-hospital mortality.
Azouz et al. (2023) [33]	I	30	41 years (craniectomy and craniotomy)	1 month	Craniectomy and Craniotomy	The in-hospital mortality rate stood at 33%. Nevertheless, no discernible disparity was observed between the two cohorts, as six patients did not survive following craniectomy, while four patients succumbed post-craniotomy.
Hutchinson et al. (2023) [34]	I	450	48.8 (Craniectomy) and 48.3 (Craniotomy)	12 months	Craniectomy and Craniotomy	The mortality rate for patients undergoing both craniectomy and craniotomy procedures was observed to be over 30% at the 12-month follow-up. There was no significant difference in mortality rates between the two groups, with 32.2% for craniectomy and 30.2% for craniotomy.

The available clinical evidence strongly suggests that patients with ASDH have lower mortality rates after undergoing craniotomy as compared to craniectomy.

Comparison of Neurological Outcomes and Postsurgical Complications Between Craniectomy and Craniotomy

Most studies used the Glasgow Coma Scale (GCS) to evaluate disease severity and patient outcomes. Lower GCS scores generally indicate more severe neurological impairment. Table 4 compares the GCS scores in patients with ASDH (upon admission) undergoing craniectomy and craniotomy.

**Table 4:** Comparing GCS scores upon admission in patients with ASDH undergoing craniectomy versus craniotomy. The studies examined in this review employed GCS scores to assess disease severity in the patient cohorts. This table presents the GCS scores of patients with ASDH upon admission. The scores are reported as mean values for the number of patients indicated within the parentheses. Rush et al. [27] did not report GCS scores. * Median, ASDH: Acute Subdural Hematoma, GCS: Glasgow Coma Scale

Authors	Craniectomy	Craniotomy
	GCS scores (number of patients)	GCS scores (number of patients)
Ruggeri et al. (2022) [20]	7.9 (44)	9.6 (50)
Ahmed et al. (2021) [21]	3.0 (518)	3 (1,852)
Tsermoulas et al. (2016) [22]	5.5 (51), 10.5 (18)	5.5 (14), 12 (16)
Vilcinis et al. (2017) [23]	5.3 (249)	9.3 (394)
Woertgen et al. (2006) [24]	5.5 (48), 11 (11), 14.5 (10)	5.5 (67), 11 (15) , 14.5 (29)
Li et al. (2012) [25]	5.5 (38), 10.5 (6), 14 (5)	5.5 (14), 10.5 (11), 14 (11)
Vankipuram et al. (2020) [26]	4.3 (59)	4.4 (56)
Shibahashi et al. (2020) [28]	7 (514)	7 (514)
Chen et al. (2011) [29]	6.3 (60)	5.9 (42)
Ahmed et al. (2020) [30]	3.0 (518)	3.0 (518)
Ran et al. (2024) [31]	5.5 (40), 10.5 (8), 14 (14)	5.5 (28), 10.5 (21), 14 (27)
Kwon et al. (2016) [32]	4 (16), 10 (10)	4 (7), 10 (13)
Azouz et al. (2023) [33]	9.4 (15)	9.6 (15)
Hutchinson et al. (2023) [34]	7.5* (222)	8* (228)

In most studies, patients undergoing craniectomy had slightly lower or comparable GCS scores than those undergoing craniotomy, suggesting that neurosurgeons utilized craniectomy for more severe cases. A few studies demonstrated negligible differences in GCS scores between the two procedures (for example, Ahmed et al. [21] and Shibahashi et al. [28]). The sample sizes varied widely, ranging from as few as 15 to over 1,800 patients, which influenced the generalizability of the findings of each study. Studies with larger sample sizes offered more reliable averages for GCS scores, such as those by Ahmed et al. [21].

Numerous studies have shown that craniectomy correlates with patients exhibiting slightly lower GCS scores upon admission, indicating a tendency to favor this intervention in cases of greater severity [20,23,25,26,33]. The presence of comparable GCS scores for both procedures across several studies suggests that additional factors, such as patient stability and the feasibility of surgical intervention, also play a significant role in determining the surgical approach employed [21,24,28,30,32].

Postsurgical complications pose a significant concern for patients undergoing brain surgery, making it essential to weigh the risks and benefits of various surgical approaches. Both craniotomy and craniectomy carry specific risks, and postoperative complications can vary widely. Common issues include elevated ICP, rebleeding, contusions, epidural hematoma, seizures, neurological deficits, reoperations, and prolonged ventilation support. The incidence and severity of these complications depend on the chosen surgical approach, highlighting the importance of closely monitoring patients during the postoperative period [20-34]. Table 5 compares postsurgical complications that occurred in patient cohorts with ASDH following craniectomy and craniotomy.

**Table 5:** Comparing postsurgical complications in patients with ASDH undergoing craniectomy versus craniotomy. The studies reviewed in this analysis focused on the complications that arose after craniectomy and craniotomy surgeries in patients. The table displays various neurological complications that patients experienced post-treatment with craniectomy or craniotomy for ASDH. The number of patients who suffered from these complications is indicated in parentheses. %: Percentage, =: Equal, >: Greater Than ARDS: Acute Respiratory Distress Syndrome, ASDH: Acute Subdural Hematoma, GCS: Glasgow Coma Scale, Hg: Mercury, ICP: Intracranial Pressure, mm: Millimeters, p: Probability Value, TRISS: Trauma and Injury Severity Score

Authors	Craniectomy	Craniotomy	Statistical significance
Ruggeri et al. (2022) [20]	Limb motility at discharge was significantly affected.	Limb motility at discharge was significantly less affected.	p = 0.020
Ahmed et al. (2021) [21]	The discharge location was home (8.5%), another hospital (4.1%), intermediate care (11.4%), and hospice (1.7%).	The discharge location was home (13.5%), another hospital (2.3%), intermediate care (12%), and hospice (1%).	p = 0.10
Tsermoulas et al. (2016) [22]	About 59% of patients had poor GCS outcomes at six months. The intracranial hypertension index was 16.6, with a standardized morbidity ratio of 0.85. Additionally, approximately 28% of patients required reoperations.	About 37% of patients had poor GCS outcomes at six months. The intracranial hypertension index was 18.6, with a standardized morbidity ratio of 0.65. Additionally, approximately 33% of patients required reoperations.	p = 0.037, p = 0.560, p = 0.727
Vilcinis et al. (2017) [23]	The mean GCS score at discharge was 1.98 ± 1.29.	The mean GCS score at discharge was 3.42 ± 1.5.	p < 0.001
Woertgen et al. (2006) [24]	Residual subdural hematoma (40%), Rebleeding subdural hematoma (4.3%), Hygroma (8.7%), ICP > 20 mmHg (73%), Days of ventilation (7.2), Days of mannitol (3), and Additional barbiturates (8.7%)	Residual subdural hematoma (61%), Rebleeding subdural hematoma (1%), Hygroma (3.6%), ICP > 20 mmHg (53%), Days of ventilation (9.4), Days of mannitol (1.9), and Additional barbiturates (8.1%).	p < 0.05
Li et al. (2012) [25]	At six-month follow-up, about 58% of patients had unfavorable GCS scores, indicating severe disability, vegetative state, and eventually death. However, 42% had favorable GCS scores, indicating a good recovery. The standardized morbidity ratio was 0.75.	At six-month follow-up, about 55% of patients had unfavorable GCS scores, indicating severe disability, vegetative state, and eventually death. However, 45% had favorable GCS scores, indicating a good recovery. The standardized morbidity ratio was 0.9.	p = 0.83
Vankipuram et al. (2020) [26]	On average, the postoperative decrease in midline shift was 3.97 ± 1.42 mm (56%). At six-month follow-up, about 37.9% of patients died. Of those who survived, based on their GCS scores, 35.3% showed a good recovery, and 26.8% still had moderate-to-severe disability.	On average, the postoperative decrease in midline shift was 4.69 ± 2.47 mm (49%). At six-month follow-up, about 45.4% of patients died. Of those who survived, based on their GCS scores, 39.2% showed a good recovery, and 15.4% still had moderate-to-severe disability.	p = 0.640
Rush et al. (2016) [27]	The discharge location was either home (20.9%) or another facility (79.1%).	The discharge location was either home (36.1%) or another facility (63.9%).	p = 0.0011
Shibahashi et al. (2020) [28]	The probability of survival per the TRISS model was 0.63.	The probability of survival per the TRISS model was 0.65.	p = 0.113
Chen et al. (2011) [29]	Two patients presented with skull bone absorption, while another exhibited skull bone sinking. Furthermore, one patient developed a skull bone infection necessitating surgical excision of the infected bone. Other complications included intracranial hematoma (40%), intracerebral hematoma (28.3%), residual subdural hematoma (0%), epidural hematoma (5%), contralateral subdural hematoma (1.7%), new hematoma (6.7%), and seizure (16.7%).	There were no complications associated with the skull bone. However, other observed complications included intracranial hematoma (47.6%), intracerebral hematoma (42.9%), residual subdural hematoma (2.4%), new hematoma (11.9%), and seizure (19%). There were no reported cases of epidural hematoma or contralateral subdural hematoma.	p > 0.05
Ahmed et al. (2020) [30]	The postoperative complications comprised ARDS in 8.1% of cases, pneumonia in 37.6% of cases, pulmonary embolism in 1.6% of cases, and sepsis in 8.1% of cases. The total duration of ventilator support was 10 days.	The postoperative complications consisted of ARDS in 12.1% of cases, pneumonia in 34.9% of cases, pulmonary embolism in 3.5% of cases, and sepsis in 6.2% of cases. The total duration of ventilation required was eight days.	p = 0.01 (only for the total duration of ventilation support)
Ran et al. (2024) [31]	Postoperative complications included pneumonia in 33.9% of cases, hydrocephalus in 12.9% of cases, and the requirement for ICP monitoring in 25.8% of cases. There were no reported incidences of subdural hematoma.	Postoperative complications included pneumonia in 15.8% of cases, hydrocephalus in 2.6%, and the need for ICP monitoring in 5.3% of cases. Re-operation surgery for residual subdural hematoma was required in 6.6% of patients.	p = 0.013, p = 0.021, p = 0.001, p = 0.04
Kwon et al. (2016) [32]	Re-operation was required for three out of 26 patients due to complications: one patient developed a subgaleal hematoma, while two patients experienced an increase in traumatic intracranial hemorrhage.	In a cohort of 20 patients, re-operation was necessary for four individuals. This included two cases of recurrent subdural hematoma and two instances of epidural hemorrhage.	p = 0.095
Azouz et al. (2023) [33]	No cases of intracranial infection or other postoperative complications were reported.	The incidence of intracranial infection was 3.3%.	p = 0.000
Hutchinson et al. (2023) [34]	The incidence of revisional surgery was notably lower, standing at 6.9%. However, wound healing was significantly delayed, predominantly attributed to a 12.2% infection rate.	The frequency of revisional surgeries increased significantly to 14.9%. However, the rate of wound healing notably improved, attributed to a substantially lower infection rate of 3.9%.	p < 0.0001, p < 0.0001

Several studies have indicated that patients undergoing craniectomy may experience higher rates of certain complications, such as infections and diffuse cerebral edema, which may manifest into intracranial hypertension if uncontrolled, as documented by Tsermoulas et al. [22] and Hutchinson et al. [34]. Statistical analyses across multiple studies demonstrate significant differences in patient outcomes between the two surgical approaches. For instance, Ruggeri et al. observed that patients who underwent craniotomy exhibited less severe limb motility impairment at discharge (p = 0.020) [20]. Additionally, Ran et al. reported considerably lower rates of pneumonia, hydrocephalus, and the need for ICP monitoring among craniotomy patients, with all findings achieving probability (p)-values below 0.05. Such results indicate that craniotomy may offer a more favorable profile regarding immediate postoperative complications, supporting its use when feasible [31].

Long-term recovery and functional outcomes further elucidate the distinctions between these two procedures. Rush et al. found that a higher proportion of patients who underwent craniotomy patients returned home post-discharge (36.1%) compared to those who underwent craniectomy (20.9%), suggesting that craniotomy may be linked to improved functional recovery. These findings underscore the implications for surgical decision-making, indicating that while craniectomy is necessary for more severe cases, craniotomy generally provides better outcomes in terms of recovery and discharge potential. Ultimately, understanding these differences is crucial for clinicians, as it enables them to select the most appropriate surgical approach tailored to the patient’s initial severity and anticipated recovery trajectory, thereby minimizing complications and enhancing overall patient outcomes [27].

Factor Influencing Selection of Craniectomy and Craniotomy

Several key elements influence the decision to choose between craniectomy and craniotomy for specific patient cohorts. These elements can be broadly categorized into four main areas: disease severity, age, geographical location, and study design. These aspects significantly impact the decision-making process of neurosurgeons and healthcare teams. Therefore, it is crucial to evaluate these considerations carefully when making an informed choice. Selecting the appropriate surgical approach should involve a comprehensive assessment of each element and other patient-specific variables to ensure optimal outcomes.

Disease severity: Most studies utilized the GCS to evaluate the severity of subdural hematoma [20-26,28-34]. Additionally, some studies employed the Injury Severity Score further to specify the injury severity [21,28-31], while others evaluated pupil reactivity to examine severity in more detail [25,26,29,31-34].

Patients with high GCS scores underwent craniotomies more frequently than those with lower GCS scores who underwent craniectomy. Furthermore, a higher number of patients with higher GCS scores underwent craniotomies than those with lower GCS scores who underwent craniectomy [20-26,28-34]. Notably, abnormal pupil findings, such as only one reactive pupil or the absence of reactivity in both pupils, were more commonly found in patients undergoing craniectomy than craniotomy [25,26,29,31-34].

Age: Several studies demonstrated that a patient’s age could help determine surgeons’ decision to select either craniectomy or craniotomy. Younger patients underwent craniectomy at a higher rate compared to their older counterparts. Additionally, younger patients under 20 years with more severe injuries underwent craniectomies [23,25-27,33]. This indicated that severely injured younger patients underwent two consecutive surgeries, as necessary for a craniectomy, and successfully recovered. In other words, younger patients who had more severe damage manifested as a significant midline shift and higher ICP warranted craniectomy and made a successful recovery compared to older patients [21,28].

Another interesting aspect regarding age and the type of surgery preferred was the cause of trauma. Younger patients who sustained severe injuries and subsequently underwent craniectomies exhibited a higher incidence of trauma resulting from suicide attempts in comparison to older patients (p = 0.039). However, there was no significant difference concerning accidental trauma between the younger and older cohorts of patients [28].

Geographical location: Among the studies reviewed, four were conducted in the U.S. [21,27,30,31], three in the United Kingdom (U.K.) [22,25,34], and one each in Italy [20], Lithuania [23], Germany [24], India [26], Japan [28], Taiwan [29], Korea [32], and Egypt [33]. The U.S.-based studies primarily focused on comparing the efficacy of craniotomies versus craniectomies for treating ASDH. Findings indicated craniotomies were more frequently performed than craniectomies in the U.S. [21,27,30,31]. However, clear evidence regarding the superiority of one procedure over the other remains inconclusive. While two studies presented strong evidence associating craniectomies with increased patient mortality [27,31], others reported no statistically significant difference in mortality rates between the two approaches [21], and one study reported no mortality [30].

The preference for craniotomies was also observed in studies outside the U.S. For instance, a German study reported a significantly higher number of patients undergoing craniotomies (111 patients) compared to craniectomies (69 patients) [24]. Similarly, in Lithuania, there was a notable disparity, with 394 patients receiving craniotomies and 249 receiving craniectomies [23]. Conversely, in the U.K., surgeons preferred craniectomies over craniotomies, with 341 patients undergoing craniectomies compared to 298 undergoing craniotomies [22,25,34]. A similar trend was noted in Taiwan, where craniectomies were favored, with 60 patients undergoing craniectomies versus 42 undergoing craniotomies [29].

Interestingly, neurosurgeons in countries such as Italy, India, Japan, Korea, and Egypt did not show a distinct preference for either craniotomy or craniectomy in treating patients with ASDH. Across these countries, a nearly equal distribution was observed, with 658 patients undergoing craniectomies and 655 patients undergoing craniotomies [20,26,28,32,33].

It was evident that a geographical factor influenced surgeons’ preference toward either craniotomy or craniectomy. In the U.S. and Germany, craniotomies were more frequently performed than craniectomies, while in the U.K., craniectomies were preferred over craniotomies. In contrast, surgeons in Italy, India, Japan, Korea, and Egypt did not exhibit a significant difference in preference for either procedure. Therefore, surgeons’ preference for craniotomy or craniectomy could have been influenced by various factors, not only the geographical location but also cultural practices and individual surgeon’s expertise, experience, and familiarity with the surgical procedure.

Study design: Studies investigating the efficacy of surgical interventions for TBIs varied significantly, with methods for determining the severity of the injury and assessing postoperative success rates differing considerably. While some studies provided detailed information about the type of craniotomy performed, such as the four-quadrant osteoplastic decompressive craniotomy [26], most did not specify the techniques. This lack of consistency in surgical techniques might contribute to differences in patient outcomes.

Injury severity was mainly determined using the GCS, although some studies used the Injury Severity Score and pupil findings for quantification. However, the studies showed inconsistent assessments of midline shift and ICP. Moreover, studies did not uniformly report medication administration, chronic medical comorbidities, intracranial hypertension index, additional operations, and postoperative residual hematomas [21,25,26,28,29,31-34].

Furthermore, the studies did not consistently specify parameters indexing risks, higher disability rates, or poor patient outcomes, limiting the ability to compare each procedure’s success rates. Good patient outcomes were determined based on GCS scores upon discharge, indicating the change from vegetative states to various levels of disability and good recovery. However, not all studies reported GCS scores upon discharge or utilized this scale, and follow-up periods varied, with some studies not specifying when the success of the surgery was determined [20,21,27,28].

The statistical analysis and conclusions drawn from these studies also varied considerably, with some studies reporting better patient outcomes with craniectomies and others reporting favorable outcomes with craniotomies in patients with severe subdural hematomas but without conducting statistical comparisons with the craniectomy arm of the study. These inconsistencies posed challenges for developing a comprehensive understanding of the efficacy of these surgical interventions for TBIs [20,23,25,26].

Determining which surgical procedure, craniotomy or craniectomy, led to better patient outcomes for individuals with severe subdural hematomas posed a challenge due to inconsistent study designs used across different studies. These studies utilized varying methods to assess injury severity, perform surgical interventions, and evaluate postoperative success rates. The heterogeneity in study designs and parameters indexing risks and severity made it difficult for surgeons to determine and rely on which surgical procedure could achieve better patient outcomes.

Discussion

This systematic review highlights crucial disparities in outcomes, procedural preferences, and complications between craniectomy and craniotomy for ASDH management. The findings provide significant insights into the advantages and disadvantages of each surgical option, empowering neurosurgeons to make well-informed decisions.

Mortality and Neurological Outcomes

One of the most notable findings of this review is the disparity in mortality rates associated with craniectomy as opposed to craniotomy. Patients undergoing craniectomy experienced higher in-hospital mortality rates, with some studies indicating rates as high as 54%, in contrast to approximately 20% in cases involving craniotomy [20,23]. This substantial difference implies that craniotomy may be associated with improved survival outcomes, mainly when the patient’s condition permits the procedure. For instance, Ruggeri et al. demonstrated that patients who underwent craniotomy exhibited enhanced neurological outcomes relative to those who received craniectomy, albeit this advantage did not achieve statistical significance. This finding highlights the potential influence of surgical choice on patient survival outcomes [20].

On the other hand, several studies demonstrated that craniectomy might be necessary for patients with lower GCS scores, where immediate decompression of ICP is critical [21,22]. This trend indicates that while craniectomy is associated with higher mortality, it may still be the preferred procedure for severe cases where rapid intervention is needed. Furthermore, patients undergoing craniotomy exhibited fewer long-term deficits in functional recovery, which aligns with findings from Woertgen et al. and Ran et al., who observed that patients undergoing craniotomy had a higher probability of returning to independent living post-discharge [24,31].

Complications and Long-Term Recovery

The review also sheds light on post-surgical complications, which vary between the two procedures. Craniectomy patients were more prone to increased ICP, infections, and the need for further surgical intervention to reinsert the bone flap, as noted by Tsermoulas et al. and Hutchinson et al. [22,34]. Craniotomy, however, was associated with specific complications, such as residual subdural hematoma and rebleeding, which posed additional challenges during recovery. The study by Chen et al. showed that craniotomy patients experienced a 42.9% rate of intracerebral hematoma, while craniectomy patients faced a slightly lower rate at 28.3%. Such findings underscore the importance of carefully selecting patients for each procedure based on individual risks and potential complications [29].

Notably, the long-term functional outcomes appeared more favorable for craniotomy patients, as they were more likely to be discharged home than to intermediate care facilities or hospice. Rush et al. found that 36.1% of craniotomy patients were discharged home, compared to only 20.9% of craniectomy patients, suggesting that craniotomy may contribute to improved post-hospitalization quality of life and independence. These results indicate that while craniectomy is beneficial in critical, life-saving situations, craniotomy may offer better outcomes for functional recovery in patients who are stable enough for this approach [27].

Factors Influencing Procedure Selection

Various factors, including disease severity, patient age, geographical location, and specific study design, influenced the decision between craniectomy and craniotomy. Disease severity, typically evaluated through the GCS, emerged as a significant determinant in selecting the surgical approach. Patients exhibiting higher GCS scores were predominantly treated with craniotomy, while those with lower scores were more often subjected to craniectomy due to the imperative nature of managing ICP [20-26,28-34]. Furthermore, abnormal pupil reactivity, such as unresponsive pupils, was noted more frequently in craniectomy patients, indicating more severe initial neurological impairment [25,26,29,31-34].

Age was another critical factor in procedure selection. Research indicated that younger patients, particularly those under 20 years of age with severe injuries, had a higher likelihood of undergoing craniectomy [23,26]. This trend may be attributed to the resilience of younger individuals, who generally demonstrate a greater capacity for recovery from additional interventions often after craniectomy, such as cranioplasty [21,28]. Additionally, in instances where younger patients sustained injuries from suicide attempts, craniectomy was more commonly performed, suggesting a potential association between trauma etiology and surgical choice [28].

Geographical location also influences surgical preferences. Studies conducted in the U.S. generally favored craniotomy over craniectomy, possibly due to variations in surgical training, demographic factors, and healthcare practices [21,27,30,31]. In contrast, craniectomy was more frequently performed in the U.K., potentially attributable to established clinical guidelines or regional preferences among neurosurgeons [22,25,34]. Other nations like Germany and Lithuania also preferred craniotomy [23,24]. Conversely, regions like Japan, Korea, and Egypt did not display a pronounced bias toward either procedure, indicating that cultural practices and surgical expertise may significantly impact procedural choices [23,24,28,32,33].

Limitations and future directions

Despite providing valuable insights, this review is limited by the heterogeneity of study designs and outcome measures, complicating direct comparisons across studies. Variability in follow-up durations, the inconsistent use of GCS, and the lack of standardized methods to measure recovery outcomes contribute to differences in reported success rates between craniectomy and craniotomy. Additionally, differences in surgical techniques, such as using four-quadrant osteoplastic decompressive craniotomy in some cases, may further confound outcome comparisons. Future studies should standardize outcome assessments and follow-up durations to facilitate more accurate evaluations of long-term efficacy.

Moreover, research into tailored surgical approaches based on patient-specific factors is warranted. Standardized guidelines that incorporate patient age, injury severity, and procedural risks are urgently needed to support neurosurgeons in selecting the most appropriate procedure for treating patients with ASDH. This approach would not only optimize patient outcomes but also minimize the risks of complications and promote faster, more effective recoveries.

## Conclusions

Craniectomy is an essential surgical intervention for critically severe ASDH cases, as it effectively facilitates rapid ICP reduction. In contrast, craniotomy typically yields better long-term outcomes for suitable candidates for the procedure. A comprehensive assessment of disease severity, patient age, and regional practices allows neurosurgeons to tailor surgical strategies to each patient's unique needs. This individualized approach improves management outcomes in ASDH and establishes a strong basis for future research on optimized and personalized treatment strategies.
